# Psychological support and postpartum depression in twin gestations: a prospective analysis using the Edinburgh Scale

**DOI:** 10.61622/rbgo/202602

**Published:** 2026-03-02

**Authors:** Magda Spinello Consul da Silva, Gustavo Yano Callado, Edward Araujo Júnior, Julio Elito

**Affiliations:** 1 Universidade Federal de São Paulo Escola Paulista de Medicina Department of Obstetrics São Paulo SP Brazil Department of Obstetrics, Escola Paulista de Medicina, Universidade Federal de São Paulo, São Paulo, SP, Brazil.; 2 Hospital Israelita Albert Einstein Faculdade Israelita de Ciências da Saúde Albert Einstein São Paulo SP Brazil Faculdade Israelita de Ciências da Saúde Albert Einstein, Hospital Israelita Albert Einstein, São Paulo, SP, Brazil.

**Keywords:** Twin pregnancy, Postpartum depression, Edinburgh Postnatal Depression Scale, Maternal mental health, Psychodynamic prenatal care

## Abstract

**Objective:**

To assess the risk of postpartum depression (PPD) in women with twin pregnancies using the Edinburgh Postnatal Depression Scale (EPDS), and to explore associations between maternal and neonatal complications and depressive symptoms during the postpartum period.

**Methods:**

This prospective, mixed-methods study was conducted at the Multiple Pregnancy Outpatient Clinic. Fourteen women with twin pregnancies received psychodynamic psychological prenatal care throughout the pregnancy-puerperal cycle. Postpartum depressive symptoms were screened using the EPDS, with scores ≥13 indicating probable PPD. Associations between EPDS scores and obstetric or perinatal variables were analyzed using Pearson correlation and contingency tables.

**Results:**

A high prevalence of depressive symptoms was observed among participants, with 28.6% screening positive for probable PPD and an additional 21.4% at mild risk. Higher EPDS scores were associated with intrapartum hemorrhage, low APGAR scores (1st and 5th <7), cesarean delivery, and neonatal intensive care unit (NICU) admission. Breastfeeding was associated with lower depressive symptom scores. Despite the presence of depressive symptoms in part of the sample, improvements in emotional domains such as anxiety and sadness were noted, suggesting a potential benefit of continuous psychodynamic support.

**Conclusion:**

Women with twin pregnancies are at increased risk of postpartum depression, particularly when compounded by obstetric and neonatal complications. Routine mental health screening and structured psychological support—such as psychodynamic prenatal care—should be considered integral components of care for women with multiple gestations. Further studies with larger samples and comparison groups are needed to refine preventive strategies.

## Introduction

Postpartum depression is a psychiatric condition that can significantly impact the mental health of the mother and her relationship with the infant(s). This condition affects between 6.5% and 20% of postpartum women worldwide.^(1)^ Diagnostic suspicion typically arises when the mother presents symptoms such as anxiety, irritability, sleep disturbances, a persistent feeling of being overwhelmed, sadness, frequent crying, insecurity regarding newborn care, concerns about harming the baby, and suicidal ideation. The main risk factor is a personal history of depression or anxiety that was not identified during pregnancy.^(2)^ Although the pathophysiology of postpartum depression remains unclear, contributing factors may include the abrupt decline in reproductive hormone levels following childbirth, genetic predispositions, and social factors.^(3)^ Among the social determinants, lack of family support, marital conflicts, history of sexual abuse, and past traumatic experiences are particularly noteworthy.

Twin pregnancies account for 2% to 3% of all births.^(4)^ However, multiple gestations are associated with a significantly higher incidence of obstetric complications, such as preterm birth, fetal growth restriction, cesarean delivery, postpartum hemorrhage, and neonatal intensive care unit (NICU) admission. These factors, combined with increased emotional burden, may significantly contribute to the psychological vulnerability of postpartum women. Accordingly, some studies have shown that twin pregnancies are associated with a higher risk of postpartum depression compared to singleton pregnancies, with this risk reaching up to 40% in recent cohorts.^(4)^

Despite the growing international literature, there is a notable scarcity of studies in Brazil that investigate the association between twin pregnancies and postpartum depression. Most available evidence comes from international cohorts, and few national studies address the psychosocial and emotional specificities of this group of women, particularly in public health contexts. This knowledge gap emphasizes the originality and relevance of the present study, which seeks to contribute to a more contextualized understanding of this mental health problem and to reinforce the need for preventive psychological strategies in prenatal and postpartum care.

Several instruments are available for the assessment of depressive symptoms during the postpartum period; however, the most widely used is the Edinburgh Postnatal Depression Scale (EPDS), developed by Cox et al.^(5)^ The EPDS is a validated and internationally recognized screening tool comprising ten items that assess the presence and severity of depressive symptoms over the previous week.

In this context, the objectives of this study were to assess the risk of postpartum depression using the EPDS in women with multiple pregnancies, and to correlate maternal and neonatal complications with postpartum depressive symptoms as measured by EPDS.

## Methods

This is a prospective study with a quantitative-qualitative approach, conducted at the Multiple Pregnancy Outpatient Clinic of the Department of Obstetrics, Paulista School of Medicine – Federal University of São Paulo (EPM-UNIFESP), grounded in the principles of Obstetric Psychology. The sample comprised twin pregnant women recruited following the first-trimester scan. Inclusion criteria were maternal age between 18 and 50 years, a minimum gestational age of 12 weeks, and heterosexual couples. Women with a history of psychiatric disorders or substance dependence were excluded.

The inclusion and exclusion criteria used in this study may create selection bias and limit the generalization capacity of the results. Patients with prior psychiatric diagnosis who were on medication were excluded from the research's population due to their higher risk of developing postpartum depression, which could act as a confounding factor and compromise the specific analysis of the relationship between twin pregnancy and depressive symptoms. This methodological decision aimed to ensure greater homogeneity of the sample but restricts the representativeness of the findings for the general obstetric population.

In addition to this, non-heterosexual couples were not included because they are treated in a specific outpatient clinic at the UNIFESP, which also affects the possibility of extrapolating the results to different family arrangements. However, this was done to ensure that these patients received the most appropriate type of care. So, these limitations should be considered when interpreting the data and can be used to highlight the need for future studies involving larger and more heterogeneous populations, allowing for more robust comparative analyses.

Participants underwent a structured psychodynamic intervention designed to provide continuous emotional support throughout the prenatal period. The intervention consisted of weekly 50-minute sessions, initiated after the diagnosis of twin pregnancy and continued up to the immediate postpartum period. Participant adherence was systematically monitored, and women who attended at least 75% of the scheduled sessions were considered active participants.

The sessions were conducted by clinical psychologists specialized in prenatal maternal mental health, working at the Multiple Pregnancy Outpatient Clinic of the Department of Obstetrics at UNIFESP. The psychodynamic intervention was based on active listening and aimed at addressing ambivalent feelings related to twin motherhood, elaborating anxieties that are typical of the gestational-puerperal period, and strengthening psychological resources to face critical situations such as preterm birth, maternal or neonatal complications, and perinatal loss.

The EPDS was administered in the immediate postpartum period to screen for depressive symptoms, between 30 and 45 days after delivery, during the inpatient stay or at the second postpartum follow-up visit. This time frame was chosen to allow for the early identification of depressive symptoms while ensuring clinical stability of the patients. The scale consists of 10 items, each scored from 0 to 3, assessing emotional states over the preceding week, yielding a total score between 0 and 30. A score of 13 or higher was considered indicative of probable postpartum depression, scores between 10 and 12 indicated mild risk, and scores below 9 were interpreted as minimal or no risk. Although no longitudinal monitoring was conducted, participants who scored above the risk threshold or endorsed item 10 (suicidal ideation) were immediately referred to the hospital's psychiatric service for specialized evaluation, ensuring ethical and safe management of identified risks.

Maternal variables (e.g., age, comorbidities, obstetric history), pregnancy-related variables (e.g., chorionicity, gestational age, mode of delivery), and perinatal outcomes (e.g., birth condition, birth weight, APGAR scores, need for NICU admission, length of hospital stay, and breastfeeding) were analyzed.

Statistical analysis was performed using the Python programming language, employing specific digital libraries such as Pandas, Numpy, and Matplotlib, along with Pearson correlation coefficients and contingency tables. The analyses aimed to identify associations between obstetric outcomes and EPDS scores, enabling a broader understanding of factors related to the development of postpartum depressive symptoms in women with twin pregnancies.

Initially, Pearson's correlation coefficient was applied to evaluate associations between EPDS scores and other clinical variables. However, it is important to highlight that Pearson's correlation is appropriate only for assessing linear relationships between continuous variables. As most clinical variables in this study were binary or categorical (e.g., preeclampsia, intrapartum hemorrhage, cesarean section, NICU admission, fetal death, breastfeeding, APGAR scores at the 1st and 5th minute < 7), the use of Pearson's correlation coefficient was not methodologically appropriate.

Alternative approaches would have been more suitable, such as effect size measures (e.g., Cohen's D) to estimate the magnitude of differences between groups, hypothesis tests (t-test or Mann–Whitney U test) to assess statistical significance, or linear regression models to evaluate associations more robustly. Given the exploratory nature of the study and its small sample size, these additional analyses were not performed, but they should be considered in future studies with larger samples.

The project was reviewed and approved by the Research Ethics Committee of UNIFESP (CAAE: 52540421.6.0000.5505).

## Results

The study sample comprised 14 twin pregnant women followed at the Multiple Pregnancy Outpatient Clinic. Maternal age ranged from 20 to 49 years, with a mean of 38.2 years. Only two participants (14.3%) conceived through assisted reproductive techniques; the remaining pregnancies were spontaneous. Regarding the mode of delivery, most participants (85.7%) underwent cesarean section. Gestational age at delivery revealed a high incidence of prematurity, with 85.7% of deliveries occurring before 37 completed weeks. Significant obstetric complications were also noted: 42.8% of women developed preeclampsia, and 21.4% experienced intrapartum hemorrhage. Additionally, 64.2% of newborns required admission to a NICU, highlighting the severity of the perinatal context (Table 1).

**Table 1:** Perinatal and delivery characteristics of twin pregnancies.

Patient	GA at delivery	Delivery mode	Breastfeeding	Preeclampsia	Growth restriction	Intrapartum hemorrhage	Newborns (Sex/APGAR scores/Weight)	NICU admission	Hospital Stay	EPDS
1	36w3d	Cesarean	Yes	Puerperal preeclampsia	No	No	1) M/9,10/2480g; 2) M/9,9/2080g	No	4 days	6
2	34w2d	Cesarean	Yes	Severe preeclampsia	No	Postpartum hemorrhage	1) F/9,9/2055g; 2) F/9,1/1700g	No	6 days	7
3	34w6d	Cesarean	No	No	–	–	–	–	–	6
4	37w0d	Cesarean	Yes	No	No	No	1) M/8,9/2580g; 2) M/9,10/2430g	1st twin only	5 days	9
5	33w1d	Cesarean	No	No	No	No	1) F/8,9/2170g; 2) F/8,9/1735g	Both	2 days (mother)	0
6	36w4d	Vaginal	Yes	No	No	Postpartum hemorrhage	1) F/9,10/2250g; 2) F/8,9/2355g	Both	4 days (mother)	3
7	32w0d	Cesarean	No	No	Selective	No	1) F/7,8; 2) F/7,8 (weight N/A)	Both	5 days (mother)	19
8	24w2d	Vaginal	No	No	TTTS	No	Stillbirths: M/605g and M/240g	No	2 days	10
9	35w0d	Cesarean	Yes	No	No	No	1) F/8,9/2270g; 2) F/8,9/2300g	No	3 days	12
10	34w5d	Cesarean	Yes	Chronic hypertension	Selective	No	1) F/8,9/1485g; 2) M/8,9/1755g	Yes	3 days	12
11	37w2d	Cesarean	Yes	No	No	Yes	–	No	2 days	5
12	34w1d	Cesarean	Yes	Preeclampsia	No	No	1) M/9,10/2225g; 2) M/9,10/1985g	Both	4 days (mother)	14
13	36w0d	Cesarean	Mixed feeding	No	No	No	1) F/6,9/2385g; 2) F/5,9/2350g	Both	2 days (mother)	24
14	31w2d	–	–	–	–	–	–	–	–	18

EPDS: Edinburgh Postnatal Depression Scale; F: female; GA: gestational age; M: male; N/A: not available; NICU: neonatal intensive care unit; TTTS: twin-to-twin transfusion syndrome

EPDS scores among participants ranged from 0 to 24 (Table 1). A total of 28.6% of postpartum women scored 13 or above, indicating probable postpartum depression. Another 21.4% scored between 10 and 12, indicating mild risk, while 50% had scores below 10, suggesting low risk. Notably, 57.1% of respondents answered "Yes, quite often" to question 10 of the EPDS ("The thought of harming myself has occurred to me"), indicating potential suicidal ideation.

Correlation analysis between maternal and delivery variables revealed associations between EPDS scores and both APGAR scores (1st and 5th minute) <7 and the occurrence of intrapartum hemorrhage, with correlation coefficients of −0.751 and −0.454, respectively (Table 2).

**Table 2:** Correlation between clinical variables and Edinburgh Postnatal Depression Scale scores.

Variable	Correlation
Preeclampsia	–0.096808
Intrauterine fetal death	–0.144205
Fetal growth restriction	0.320210
Gestational age at delivery	–0.148622
Cesarean delivery	0.248033
Intrapartum hemorrhage	–0.454251
APGAR scores < 7	–0.751075
NICU admission – Twin 1	0.219268
NICU admission – Twin 2	0.116083
Breastfeeding	0.037234

NICU: neonatal intensive care unit

Contingency analysis showed that among the women who had vaginal deliveries, 33.3% (1/3) screened positive for probable postpartum depression, whereas 50% (5/10) of those who underwent cesarean section also scored above the EPDS threshold. Although the proportion of depressive symptoms was higher in the cesarean group, the difference between delivery modes was not statistically significant, likely due to the small sample size.

The relationship between breastfeeding and postpartum depression was also explored: among those who breastfed, 44.4% (4/9) screened positive for probable depression, compared to 33.3% (1/3) of those who did not breastfeed. Breastfeeding was not a protective effect for postpartum depression in this small sample, where depressive symptoms were observed in both groups.

Finally, individual responses to the EPDS items revealed that, despite the presence of depressive symptoms in part of the sample, notable improvements were reported in domains such as anxiety, guilt, sadness, and crying when compared to the prenatal period, suggesting a possible positive impact of the psychological support provided throughout the pregnancy-puerperal cycle.

Although alternative statistical methods (such as effect size measures, hypothesis testing, or regression models) would have been more appropriate for categorical variables, it was not feasible to modify Table 2 due to the small sample size and the exploratory nature of this study. Reanalyzing the data with more robust statistical tests would likely require a larger cohort to ensure adequate statistical power and validity of the results. Therefore, the current table should be interpreted with caution, as an exploratory description rather than a definitive inferential analysis.

## Discussion

This study revealed a high prevalence of depressive symptoms among women with twin pregnancies in the postpartum period, highlighting the emotional vulnerability associated with this obstetric condition. Psychological distress was frequently observed in women exposed to perinatal complications such as cesarean delivery, NICU admission, and obstetric hemorrhage. These findings may suggest that both the inherent physiological demands of twin gestation and the associated adverse outcomes may play a role in triggering or exacerbating postpartum emotional distress. Notably, the psychodynamic prenatal support offered throughout the pregnancy-puerperal cycle may have contributed to some degree of emotional regulation, particularly in domains related to anxiety, guilt, and sadness.

Several recent large-scale cohort and registry-based studies have confirmed that women with twin pregnancies are at increased risk of developing postpartum depression compared to those with singleton pregnancies. A Japanese nationwide cohort study reported a significantly higher prevalence of postpartum depression at six months postpartum among women with multiple pregnancies, although the difference at one month postpartum was not statistically significant.^(6)^ Similarly, a Danish study found a 24% higher cumulative risk of postpartum depression among mothers of twins, with the hazard peaking around two months postpartum.^(4)^ A Canadian population-based cohort^(7)^ and earlier U.S. data from the ECLS-B cohort^(8)^ also confirmed a higher incidence of maternal mental illness or depressive symptoms in mothers of twins.

Despite this consistent trend, some studies suggest that contextual factors may influence outcomes. For example, a U.S. administrative database study found no overall difference in the prevalence of severe psychiatric morbidity between twin and singleton deliveries; however, among women with comorbidities, severe maternal morbidity, or low income, the risk was significantly higher for twin deliveries.^(9)^ An Italian cohort study with structured psychological support found no significant difference in EPDS scores between mothers of twins and singletons, although both groups exhibited a high prevalence of depressive symptoms and suicidal ideation—suggesting that adequate mental health support may mitigate risk.^(10)^ Overall, most studies converge in showing an increased relative risk for postpartum depression in twin pregnancies, particularly during the first 6–12 months postpartum and among women with preexisting vulnerabilities.^(4,6,7,10)^

Consistent with previous research, the present study found that maternal complications—particularly preeclampsia and intrapartum hemorrhage—were associated with higher EPDS scores in the postpartum period. These findings align with evidence from umbrella reviews and large cohort studies demonstrating that preeclampsia, gestational diabetes, anemia, and emergency cesarean section are among the most robustly associated maternal risk factors for postpartum depression, especially when complications occur cumulatively during pregnancy and delivery.^(11-13)^ The emotional and physical strain imposed by unexpected or high-risk obstetric events may intensify psychological distress during the transition to motherhood, particularly in the context of a twin pregnancy, which is already a high-burden scenario.

In addition, our results reinforce the association between neonatal complications and increased risk of maternal depressive symptoms. The high rate of NICU admission among the newborns in our sample was significantly correlated with elevated EPDS scores, consistent with previous studies linking preterm birth, low birth weight, NICU stay, and congenital anomalies to higher postpartum depression risk.^(14-16)^ Although we did not evaluate infant sex or congenital anomalies specifically, the literature suggests that even the perception of neonatal vulnerability may contribute to maternal anxiety and depressive symptoms, particularly when combined with feelings of helplessness or guilt about the birth process.^(17,18)^ These findings underscore the importance of comprehensive perinatal care that integrates psychological assessment and support, especially in the presence of perinatal complications.

Although the evidence base for psychodynamic psychotherapy in the prevention of postpartum depression remains limited compared to cognitive-behavioral or interpersonal therapies, available studies suggest it may offer meaningful benefits, particularly in settings where long-term therapeutic rapport and emotional elaboration are prioritized. The psychological prenatal care offered in the present study followed a psychodynamic framework, emphasizing continuous support throughout pregnancy and the postpartum period. While this approach has not been as rigorously evaluated in large randomized trials, recent systematic reviews suggest it may reduce depressive symptoms across different settings and formats.^(19)^ The observed improvements in emotional domains such as anxiety, guilt, and sadness in our participants –– despite the presence of clinical depression in some cases –– may reflect the potential of this model to foster resilience and mitigate psychological distress in high-risk pregnancies.

These findings support growing international consensus that structured psychological interventions initiated during pregnancy can reduce the incidence and severity of postpartum depression. Meta-analyses and large-scale reviews—particularly those informing the U.S. Preventive Services Task Force—highlight that counseling interventions such as cognitive-behavioral therapy and interpersonal therapy are associated with up to 50% relative risk reduction for perinatal depression, especially in high-risk populations.^(20,21)^ Although the intervention model used in our study differs in structure and modality, its integration into routine prenatal care represents a promising step toward comprehensive, multidisciplinary support. Future studies should explore the comparative effectiveness of psychodynamic and structured approaches in diverse populations, especially those with increased vulnerability due to twin gestation or obstetric complications.

This study has several limitations. First, the sample size was small, which limits the generalizability of the findings and the statistical power to detect associations. Second, the absence of a control group of singleton pregnancies prevents direct comparison of postpartum depression risk between different gestational types. Third, the use of the EPDS as a screening tool, although widely validated, does not constitute a clinical diagnosis and may be influenced by subjective interpretations. Furthermore, the sociodemographic characteristics of the sample, including its restriction to heterosexual couples and exclusion of women with preexisting psychiatric disorders, may limit the external validity and representativeness of the results. Finally, the cross-sectional application of the EPDS in the early postpartum period did not allow for longitudinal monitoring of the evolution of symptoms.

The data, despite the small sample size, suggest an increased prevalence of postpartum depressive symptoms among women with twin pregnancies, particularly in the presence of obstetric complications. However, it is essential to avoid broad generalizations, given the non-randomized design and specific inclusion and exclusion criteria of the study. Moreover, the absence of a control group and the cross-sectional nature of the postpartum assessment limit causal inference.

Several methodological limitations must be considered, including small sample size, selection bias due to the exclusion criteria, and lack of longitudinal follow-up, which may have underestimated depressive symptoms emerging later in the postpartum period. Despite these constraints, the study provides clinically relevant insights, particularly regarding the protective role of psychological prenatal care with a psychodynamic focus, which may help reduce emotional distress and strengthen the mother–infant bond.

Additionally, this discussion should consider the structural context of the Brazilian Unified Health System (SUS), where systematic psychological support during pregnancy is still scarce. Integrating low-cost psychosocial strategies, such as group interventions, brief counseling sessions during routine prenatal care visits, and collaborative care involving mental health professionals, obstetricians, and social workers, could expand access to emotional support for women experiencing high-risk pregnancies.

One methodological limitation of this study lies in the use of Pearson's correlation coefficient with categorical variables. Since Pearson's correlation coefficient is not suitable for such data, the results of these correlations should be interpreted with caution. Future research with larger samples should employ more appropriate statistical approaches, such as effect size estimation, hypothesis testing, or regression models, to provide a more accurate assessment of the associations between clinical variables and depressive symptom scores.

Future research with larger, more heterogeneous samples and longitudinal designs is necessary to provide more robust evidence and support the development of comprehensive perinatal mental health strategies tailored to the needs of twin pregnancies.

Despite these limitations, the study has relevant strengths. It is one of the few investigations in Brazil to prospectively explore postpartum depression in women with twin pregnancies, combining a validated screening tool with psychological follow-up grounded in Obstetric Psychology. The integration of qualitative and quantitative perspectives, as well as the use of statistical methods to correlate emotional outcomes with maternal and neonatal variables, enriches the clinical and academic relevance of the findings.

## Conclusion

In conclusion, the data from this study suggest an increased risk of postpartum depression among women with twin pregnancies, particularly in the presence of adverse obstetric and neonatal outcomes such as intrapartum hemorrhage, prematurity, low APGAR scores, and high rates of cesarean section. These findings are consistent with previous research indicating greater emotional vulnerability in this population. The results highlight the importance of routine psychological screening and structured mental health support during prenatal and postnatal care, especially for high-risk pregnancies. Psychodynamic prenatal care may play a protective role in mitigating emotional distress and supporting maternal adaptation. However, these conclusions must be interpreted with caution. The small sample size and absence of a control group limit the generalizability of the findings. Further research with larger, more diverse samples is needed to strengthen the evidence and inform more robust clinical guidelines.

## Data Availability

The research data are described in the article presented.
